# Supplementary material for: Four ways blue foods can help achieve food system ambitions across nations
Source: Nature. 2023 Feb 22;616(7955):104–12. doi: 10.1038/s41586-023-05737-x (PMC10076219; doi:10.1038/s41586-023-05737-x)
Supplement: Supplementary file 1 — This file contains Supplementary Tables 1 and 2 and References. [file 41586_2023_5737_MOESM1_ESM.pdf]

nature portfolio
https://doi.org/10.1038/s41586-023-05737-x

Supplementary information

Four ways blue foods can help achieve food system ambitions across nations

In the format provided by the
authors and unedited

Supplementary Tables

Supplementary Table S1. Full description of each variable and data source, the cut-off value, and the methodology used for setting cut-off values. Plots of each variable are provided in Extended Data Figure 1. Grey rows indicate which policy objective is in focus. 'Leveraged expertise' indicates the disciplinary competencies used to assess the plausibility of selected cut-offs.

Variable	Variable description	Data source	Cut-off value and inclusion criteria	Cut-off decision methodology	Leveraged expertise
**Policy objective: Reducing blue food sensitive nutrient deficiencies**					
Nutrient insufficiency (omega-3)	Estimated proportion of population with an inadequate intake of omega-3, represented as summary exposure values (SEV)<br><br>SEVs are a measure of a population's exposure to a risk factor that takes into account the extent of exposure by risk level and the severity of that risk's contribution to disease burden. Zero represents a situation where no excess risk for a population exists, and 1 is when the population is at the highest level of risk. SEVs are reported on a scale from 0% to 100% to emphasize that it is risk-weighted prevalence[116].	[117]	10% (above 10 included)	No literature exists to guide a specific cut-off for when the proportion of population lacking omega-3 exceeds a value where it should be considered a significant problem. Arguable any deficiency is a concern, yet virtually all countries have some inhabitants lacking intake. Based on a clear natural break in the distribution at around 10%, this was chosen as the cut-off.	Nutrition, Epidemiology
Nutrient insufficiency (vitamin B12)	Estimated proportion of population with an inadequate intake of vitamin B12, represented as summary exposure values (SEV).	[117]	10% (above 10 included)	No literature exists to guide a specific cut-off for when the proportion of population lacking vitamin B12 exceeds a value where it should be considered a significant problem. Arguable any deficiency is a concern, yet few countries have no inhabitants lacking intake. For consistency, the 10% cut-off used for omega-3 was applied for B12 as well.	Nutrition, Epidemiology
Potential blue food availability	Measured as the total availability of blue foods available to a nation, based on domestic production and imports. Calculated as the sum of production and imports. Exports were not deducted, because we were interested in capturing the potential availability, should decisions be made to divert volumes currently exported to domestic consumption.<br><br><u>Production</u>: for each country, average production (2006-2016) was divided by the average population (2006-2016). Aquatic plants and algae excluded.<br><u>Imports</u>: all codes in category 03 (except 0301.11 , 0301.19; Ornamental fish) were averaged across 3 years, 2015, 2016, 2017 (years chosen to match latest population data from FishstatJ) divided by the average population.	[118]	Sum of production and imports equal 8 kg/cap/year (above 10 included)	No literature exists to determine when supply of a given commodity is high or low.<br><br>8kg/cap/yr represents 50% of the average global per cap blue food availability (excluding China) (16 kg/cap/year[119].<br>This was seen as an acceptable level for when blue foods can be deemed to be reasonably available.	Nutrition, Fisheries trade

Variable	Variable description	Data source	Cut-off value and inclusion criteria	Cut-off decision methodology	Leveraged expertise
**Policy objective: Reducing cardiovascular disease risk**					
Red meat consumption	Reported red meat consumption in grams per capita per day. Includes data on "Bovine Meat", and "Mutton & Goat Meat" and "Pigmeat". Averaged across the latest three years of data (2009-2011). Converted from raw weight to cooked weight with conversion factor (0.746).	[120]	50 g/cap/day (above 50 included)	Based on the average recommended daily allowance of dietary guidelines of 29 countries (Springmann et al 2020-BMJ)	Nutrition, Epidemiology, Planetary health
Cardiovascular disease risk	Measured as disability adjusted life years (DALYs) per capita. Data used includes DALY for all ages, for persons (men and women combined) from 2019.	[121]	0.05 year/cap (above 0.05 included)	Based on break in data (when ordered by rank). Countries included using this cut-off were also cross-checked with WHO reports to make sure most nations consistently cited by WHO as grappling with high rates of heart disease were captured.	Nutrition, Epidemiology,
Potential blue food availability	See above for description				
**Policy objective: Reducing environmental footprints of food consumption and production**					
Ruminant meat consumption	Reported ruminant meat consumption in grams per capita per day. Includes data on "Bovine Meat", and "Mutton & Goat Meat". Averaged across the latest three years of data (2009-2011). Converted from raw weight to cooked weight with conversion factor (0.746).	[120]	7 g/cap/day (above 7 included)	Based on the average recommended daily allowance of the EAT-Lancet[122]	Nutrition, Epidemiology, Planetary health
Potential blue food availability	See above for description				
**Policy objective: Safeguarding food system contributions under climate change**					
Blue food contribution to employment	Represents an aggregate measure of number of people employed in marine and inland fisheries, and aquaculture sector.<br>Calculated as the total number of people employed in the blue food sector as a % of total workforce.	[123-126]	1% (above 1% included)	No scientific consensus was found for when a sector can be considered to contribute significantly to a nation's employment. However, the covid pandemic raised global average unemployment by 1% (from 5.4% to 6.5%). This has been considered a drastic shock to employment. We therefore set the cut-offs for when employment contribution of blue foods can be considered significant as 1%	General knowledge of fisheries and aquaculture and their contribution to national economies
Blue food generated export value	Calculated as blue food export value in 2019 and expressed as a proportion of GDP (most recently available value)	Export value[125]<br><br>GDP[127]	3% (above 3% included)	No literature exists to determine when a commodity contributes significantly to GDP. We therefore use a natural break in the rank ordered data	General knowledge of fisheries and aquaculture and their contribution to national economies
Blue food nutritional contribution	Assessment of the reliance of a nation on blue foods for nutrition. Calculated as a proportion of blue foods contributing to total animal protein consumption in a	[128]	20% (above 20 included)	Based on literature stating 20% as the threshold by which a country becomes "fish reliant"[119, 129]	Nutrition, Epidemiology, as well as general knowledge of fisheries and aquaculture and their contribution to

	nation. Higher values indicate higher reliance.<br>For each country mean of individual animal food categories in FAO New Food Balance sheets were averaged across the most recent years (2014-2018). Protein consumption values (based on supply quantity) were extracted (g/capita/day) and summed into two categories; aquatic foods and terrestrial foods. Aquatic foods include: "Fish, Body Oil",Fish, Liver Oil","Freshwater Fish","Demersal Fish","Pelagic Fish","Marine Fish, Other","Crustaceans","Cephalopods","Molluscs, Other","Meat, Aquatic Mammals","Aquatic Animals, Others"<br>Terrestrial foods include: "Bovine Meat","Meat, Other","Mutton & Goat eat","Pigmeat","Poultry Meat","Milk - Excluding Butter","Butter, “Ghee","Cream",Offals, Edible","Eggs","Fats, Animals, Raw"				national economies
Climate hazard	National level climate hazard score, ranging from 0-100, that integrates multiple environmental threats (such as changing temperature, sea level rise, ocean acidification) to marine and freshwater fisheries, aquaculture and supply chains based on present-day production portfolios. Values are based on a high-emissions (SSP5-8.5) scenario and averaged over the years 2040-2060	[130]	50 (above 50 included)	A score of 0-25 means ‘low hazard’, 25-50 means ‘medium hazard’, 50-75 means ‘high hazard’, and 75-100 means ‘very high hazard’. This analysis set the cut-off to include all countries scored as ‘high’ and ‘very high’ hazard	Fisheries oceanography, Climate change impacts on food systems

Supplementary Table S2. Description of criteria used for classification analysis to match countries to each policy objective. Countries were classified to one of the four categories ‘highly relevant, ‘relevant’, ‘less relevant, and ‘missing data’, depending on how fully they matched the stipulated criteria outlined for each policy objective (Table S1). These criteria represent the conditions when policy objectives are hypothesized to be relevant. R code used for Boolean analysis is available here https://github.com/emmywas/BFA_Policy_analysis

BF=blue food, *countries with missing data for blue food availability were included in these categories.

Note that variables ‘blue food availability’ and ‘climate hazard score’ primarily differentiate between countries for which the policy is highly relevant, vs those where it is just relevant. The logic is that for a country with, for example, inadequate intake of B12 or omega-3, low blue food availability does not render the policy irrelevant. It simply means the conditions for most easily implementing it, given current conditions, exists in ‘highly relevant’ countries, while in ‘relevant’ countries blue food may need to be sourced.

**Degree of policy relevance**	**Reducing blue food sensitive deficiencies**		**Reducing cardiovascular disease risk**	**Reducing environmental footprints of food consumption and production**	**Safeguarding food system contributions under climate change**
	**omega-3**	**Vitamin B12**			
Highly relevant	(High insufficient intake of vitamin omega-3 **AND** BF availability above the cut-off	(High insufficient intake of vitamin B12 **AND** BF availability above the cut-off	Red meat consumption is high **AND** cardiovascular disease risk is high **AND** BF availability is above the cut-off	Ruminant meat consumption is high **AND** BF availability is above the cut-off	(The BF sector is important due to employment **OR** export revenue **OR** high nutritional contribution) **AND** country faces a high climate hazard
Relevant	(High insufficient intake of vitamin omega-3 **AND** BF availability is below the cut-off*	(High insufficient intake of vitamin B12 **AND** BF availability is below the cut-off*	Red meat consumption is high **AND** cardiovascular disease risk is high **AND** BF availability is below the cut-off*	Ruminant meat consumption is high **AND** BF availability is below the cut-off*	(The BF sector is important due to employment **OR** export revenue **OR** high nutritional contribution) **AND** the country does not face a high climate hazard
Less relevant	Low incidence of insufficient intake of vitamin omega-3	Low incidence of insufficient intake of vitamin B12	Red meat consumption is low **OR** cardiovascular disease risk is low.	Ruminant meat consumption is below the cut-off	The BF sector is not important for employment, export revenue, or high nutritional contribution (i.e., all variables below threshold).
Missing data	Data not available for intake of vitamin omega-3	Data not available for intake of vitamin B12	Data not available for red meat consumption or cardiovascular disease incidence variables	Data not available ruminant meat consumption	Data not available for employment, export revenue, or nutritional contribution

References

116. Institute for Health Metrics and Evaluation. Terms Defined. Available at: https://www.healthdata.org/terms-defined/s. (Accessed: 1st April 2022)
117. Golden, C. D. *et al.* Aquatic Foods for Nourishing Nations. *Nature* **598**, 315-320 (2021).
118. FAO. *Fishery and Aquaculture Statistics. Global capture production 1950 - 2019 (FishstatJ)*. (2021).
119. FAO. *The State of World Fisheries and Aquaculture 2020. Sustainability in action. The State of World Fisheries and Aquaculture 2020. In brief* (FAO, 2020). doi:10.4060/CA9231EN
120. Smith, M. R., Micha, R., Golden, C. D., Mozaffarian, D. & Myers, S. S. Global expanded nutrient supply (GENuS) model: A new method for estimating the global dietary supply of nutrients. *PLoS One* **11**, (2016).
121. WHO. Global health estimates: Leading causes of DALYs. Available at: https://www.who.int/data/gho/data/themes/mortality-and-global-health-estimates/global-health-estimates-leading-causes-of-dalys. (Accessed: 3rd September 2021)
122 Willet, W. *et al.* Food in the Anthropocene: the EAT–Lancet Commission on healthy diets from sustainable food systems. *The Lancet* **393**, 447-492 (2019)

123. Funge-Smith, S. & Bennett, A. A fresh look at inland fisheries and their role in food security and livelihoods. *Fish Fish.* **20**, 1176–1195 (2019).
124. Teh, L. C. L. & Sumaila, U. R. Contribution of marine fisheries to worldwide employment. *Fish Fish.* **14**, 77–88 (2013).
125. FAO. FAO Yearbook. Fishery and Aquaculture Statistics 2018.
126. ILO. ILOSTAT labour statistics. (2020). Available at: https://ilostat.ilo.org/. (Accessed: 3rd September 2021)
127. World Bank. World Development Indicators | DataBank. (2012). Available at: https://databank.worldbank.org/reports.aspx?source=world-development-indicators. (Accessed: 3rd September 2021)
128. FAO. FAOSTAT Food Balances. Available at: http://www.fao.org/faostat/en/#data/FBS. (Accessed: 3rd September 2021)
129 Golden, C. D., *et al.* Nutrition: Fall in fish catch threatens human health. *Nature* **534,** 317-320 (2016)
130. Tigchelaar, M. *et al.* Compound climate risks threaten aquatic food benefits. *Nat. Food* **2**, 673-682 (2021).
